# Bacteriophage Therapy for the Prevention and Treatment of Fracture-Related Infection Caused by Staphylococcus aureus: a Preclinical Study

**DOI:** 10.1128/spectrum.01736-21

**Published:** 2021-12-15

**Authors:** Jolien Onsea, Virginia Post, Tim Buchholz, Hella Schwegler, Stephan Zeiter, Jeroen Wagemans, Jean-Paul Pirnay, Maya Merabishvili, Matteo D’Este, Stijn G. Rotman, Andrej Trampuz, Michael H. J. Verhofstad, William T. Obremskey, Rob Lavigne, R. Geoff Richards, T. Fintan Moriarty, Willem-Jan Metsemakers

**Affiliations:** a Department of Trauma Surgery, University Hospitals Leuven, Leuven, Belgium; b Department of Development and Regeneration, KU Leuven, Leuven, Belgium; c AO Research Institute Davos, Davos, Switzerland; d Laboratory of Gene Technology, KU Leuven, Leuven, Belgium; e Laboratory for Molecular and Cellular Technology, Queen Astrid Military Hospital, Brussels, Belgium; f Center for Musculoskeletal Surgery, Charité-Universitätsmedizin Berlin, Freie Universität Berlin, Humboldt-Universität zu Berlin, and Berlin Institute of Health, Berlin, Germany; g Trauma Research Unit Department of Surgery, Erasmus MC, University Medical Center Rotterdam, Rotterdam, the Netherlands; h Department of Orthopaedic Surgery and Rehabilitation, Vanderbilt University Medical Center, Nashville, Tennessee, USA; Institut Pasteur

**Keywords:** bacteriophages, *Staphylococcus aureus*, fracture-related infection, implant, rabbit

## Abstract

Although several studies have shown promising clinical outcomes of phage therapy in patients with orthopedic device-related infections, questions remain regarding the optimal application protocol, systemic effects, and the impact of the immune response. This study provides a proof-of-concept of phage therapy in a clinically relevant rabbit model of fracture-related infection (FRI) caused by Staphylococcus aureus. In a prevention setting, phage in saline (without any biomaterial-based carrier) was highly effective in the prevention of FRI, compared to systemic antibiotic prophylaxis alone. In the subsequent study involving treatment of established infection, daily administration of phage in saline through a subcutaneous access tube was compared to a single intraoperative application of a phage-loaded hydrogel and a control group receiving antibiotics only. In this setting, although a possible trend of bacterial load reduction on the implant was observed with the phage-loaded hydrogel, no superior effect of phage therapy was found compared to antibiotic treatment alone. The application of phage in saline through a subcutaneous access tube was, however, complicated by superinfection and the development of neutralizing antibodies. The latter was not found in the animals that received the phage-loaded hydrogel, which may indicate that encapsulation of phages into a carrier such as a hydrogel limits their exposure to the adaptive immune system. These studies show phage therapy can be useful in targeting orthopedic device-related infection, however, further research and improvements of these application methods are required for this complex clinical setting.

**IMPORTANCE** Because of the growing spread of antimicrobial resistance, the use of alternative prevention and treatment strategies is gaining interest. Although the therapeutic potential of bacteriophages has been demonstrated in a number of case reports and series over the past decade, many unanswered questions remain regarding the optimal application protocol. Furthermore, a major concern during phage therapy is the induction of phage neutralizing antibodies. This study aimed at providing a proof-of-concept of phage therapy in a clinically relevant rabbit model of fracture-related infection caused by Staphylococcus aureus. Phage therapy was applied as prophylaxis in a first phase, and as treatment of an established infection in a second phase. The development of phage neutralizing antibodies was evaluated in the treatment study. This study demonstrates that phage therapy can be useful in targeting orthopedic device-related infection, especially as prophylaxis; however, further research and improvements of these application methods are required.

## INTRODUCTION

The use of fracture fixation devices has had an enormously positive impact on patient care, with shorter hospital stays and a more rapid return to function. However, a major drawback to this technological advance is the risk of developing device-related infections (1). The incidence of fracture-related infection (FRI) after internal fracture fixation ranges between 1 and 2% for closed fractures, and up to 30% for open fractures (2–5). While a variety of microorganisms can be involved, staphylococci such as Staphylococcus aureus are the most common causative pathogens (6, 7). Their ability to adhere to foreign objects such as fracture fixation devices, and to form antibiotic tolerant biofilms, contributes to their pathogenicity (7). As a consequence, treatment is challenging and consists of both surgical debridement and long-term antibiotic treatment. In most cases, the implant needs to be removed as the biofilm mode of growth renders conventional antibiotic treatments ineffective (6). Moreover, in chronic or late-onset cases there is a relatively high risk of treatment failure, leading to additional surgical revisions, longer length of hospital stay and increased morbidity (8). In case of antibiotic resistant pathogens, such as methicillin-resistant S. aureus (MRSA), treatment options may be even further restricted, and the risk of treatment failure may even be higher (9).

For the above-mentioned reasons, alternative antimicrobial strategies are being investigated, including bacteriophage (phage) therapy. The therapeutic potential of phages to support treatment of challenging cases of orthopedic device-related infections has been demonstrated in a number of case reports and series over the past decade (10–16). However, many unanswered questions remain concerning the optimal application protocol (17). Phage therapy is most often applied locally to patients with a musculoskeletal infection, whereby phage suspensions are prepared in a buffered solution and applied directly into the wound (10, 12–14, 18). This approach generally requires repeated daily dosing by an external draining system for a period of up to 10 days postoperatively. This places an additional burden on patients as they must remain in the hospital for a longer period of time with a percutaneous drain, which is prone to superinfection (12). Furthermore, preclinical models have shown that the induction of phage-neutralizing antibodies is common and can have detrimental effects on the local phage titer when treatment is prolonged (19, 20). However, it is not clear how these laboratory observations translate to phage treatments in patients. Furthermore, given the variety of phage application protocols and the diversity of phages, there is no universal predictive model of antibody induction by phages. It has been suggested that encapsulation of phages in a carrier may protect them from the adaptive immune system (19). Furthermore, analogous to antibiotic delivery vehicles such as antibiotic-loaded collagen sponges, the development of a carrier that allows a sustained release of phages would be an important clinical improvement reducing the burden on the patient, reducing risk of superinfection, and requiring fewer bedside interventions (21, 22). This may be a clinically attractive approach, although to date, only one case report has been published in the literature (15).

The aim of this study was to provide a proof-of-concept of phage therapy in a clinically relevant rabbit model of FRI caused by S. aureus, whereby the phage was applied as prophylaxis in a first phase, and as treatment of an established infection in a second phase. Phage therapy was applied in a prophylaxis mode in saline, and in a treatment of established infection mode as conventional delivery of repeated doses of phage suspension in saline through a subcutaneous access tube, or a single application in a phage-loaded hydrogel.

## RESULTS

### Animal welfare.

**FIG 1:**
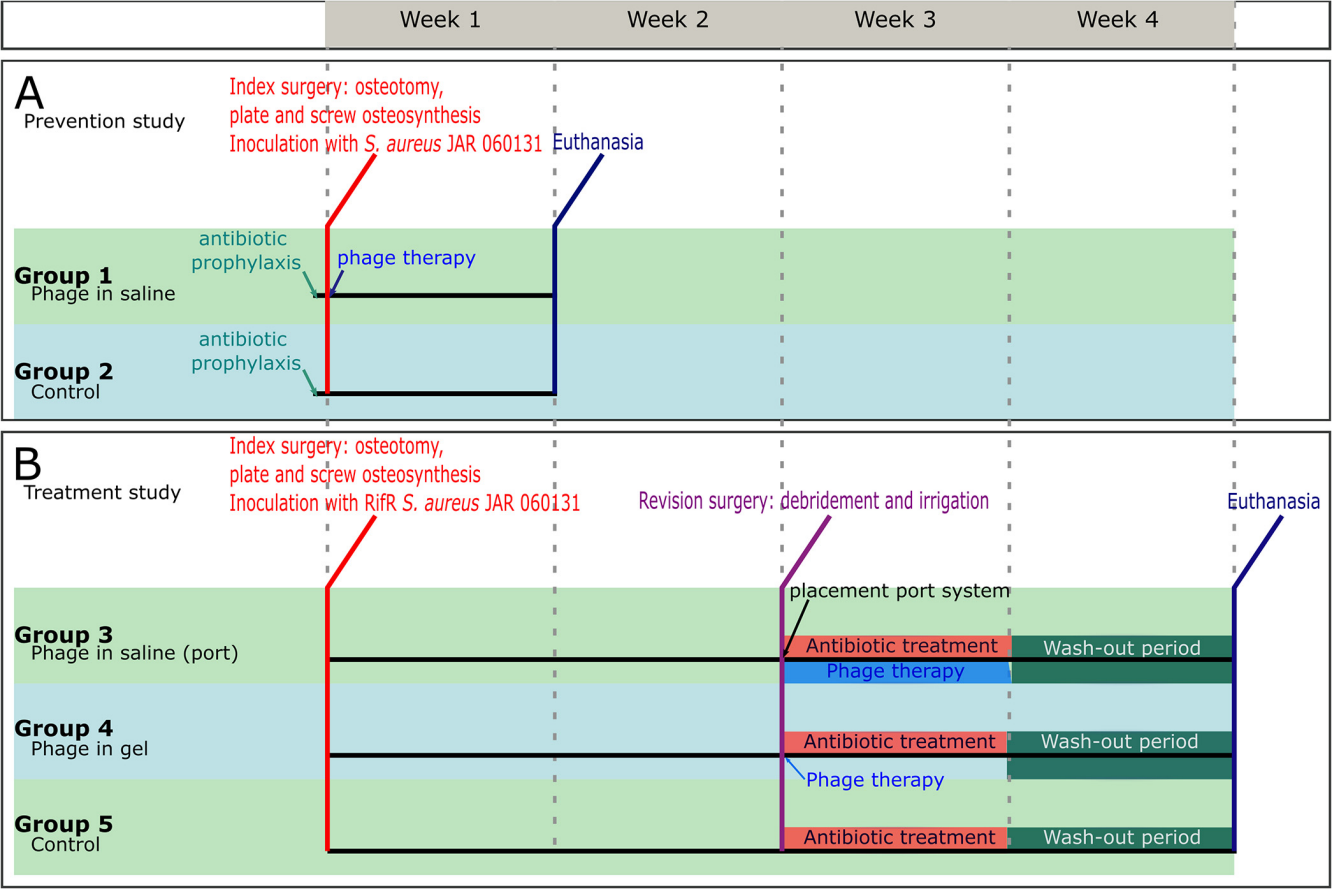
Study design and group allocations. A. Study design for the use of phage therapy in a prevention setting. In Group 1, phage in saline was applied intraoperatively. B. Study design for the use of phage therapy in a treatment setting. In Group 3, a subcutaneous access tube was placed during revision surgery in order to administer phage in saline two times per day for a week. In Group 4, a phage-loaded hydrogel was applied locally on the plate. In all treatment groups, antibiotic treatment consisted of nafcillin (given subcutaneously) and rifampicin (given orally). During the wash-out period the animals did not receive antibiotic or phage treatment.

In total, 42 animals were included in this study. Two rabbits died during induction of anesthesia for the index surgery due to unknown reasons. Two animals in the treatment study were culture-negative at revision surgery (*n* = 1, Group 3 and *n* = 1, Group 4), which made it impossible to assess the effect of the intervention on the infection and were, therefore, euthanized. The group allocation and study design for the prevention and treatment study are presented in Fig. 1. One animal (Group 4) died overnight and was found dead in its cage on the final study day and retained within the study. Three animals (2 animals from Group 3, 1 animal from Group 4) were euthanized 1 day prior to the scheduled euthanasia due to acute diarrhea and were retained within the study population. Parasitological analysis and bacteriology of fecal samples did not yield any identifiable cause of the acute diarrhea. All other animals tolerated surgery and survived the complete study duration. After replacement surgeries, a total of 38 rabbits were included in the final analysis, with seven to eight rabbits per group.

### Prevention study.

**FIG 2:**
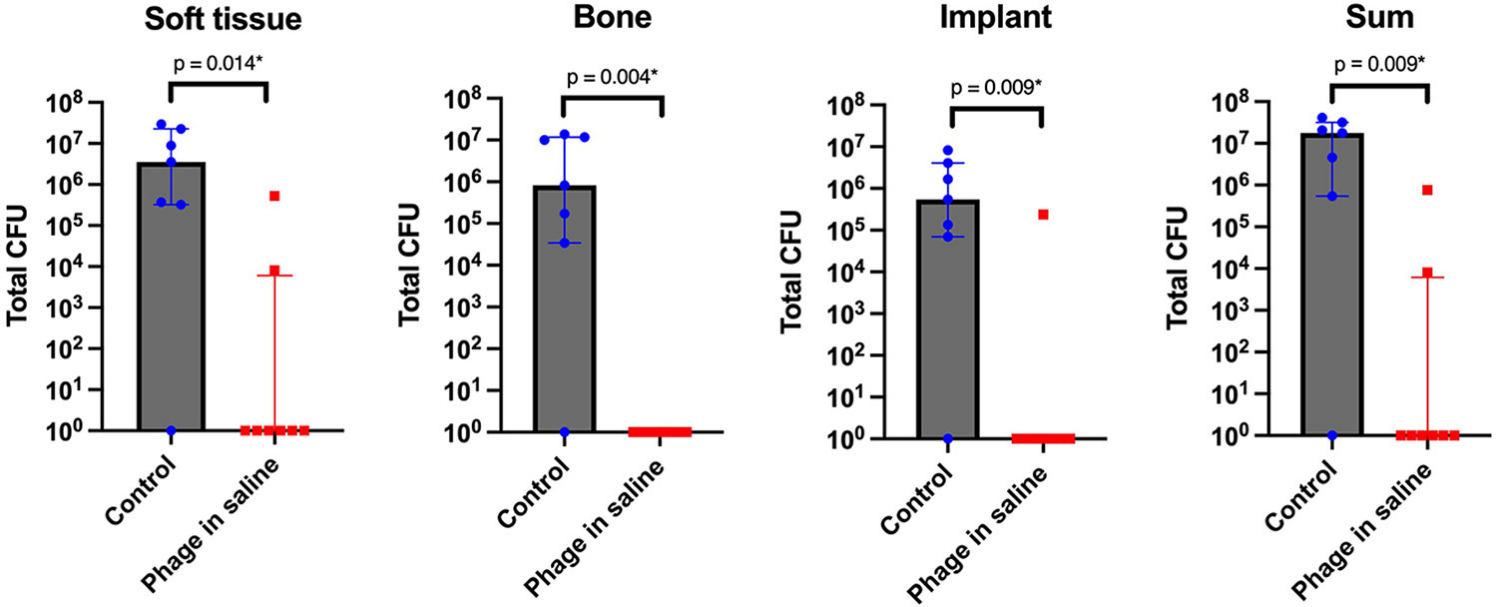
Quantitative bacteriological evaluation of soft tissue, implant and bone, and the sum of all three is shown for the control (systemic antibiotic only) animals (*n* = 7) and the phage-treated animals (*n* = 8) that received phage therapy soon after inoculation as a prevention measure. The median and interquartile range are shown for each group. The asterisk indicates a statistically significant result (*P* < 0.05). CFU, colony forming units.

The quantitative bacteriological results from the prevention study are displayed in Fig. 2. Compared to the control group (Group 2), the group receiving phage in saline (Group 1) showed a significant reduction in bacterial load at all sampled locations (soft tissue, implant, bone and overall). The infection was eradicated in six out of eight animals receiving phage in saline. In the control group, infection was eradicated in only one out of seven. The isolated pathogens in the group that received phage in saline (Group 1) remained susceptible to ISP (intravenous staphylococcal phage, see Materials and Methods–Bacteriophage therapy), with a median EOP (efficiency of plating, see Materials and Methods–Phage susceptibility tests) of 1.60 (P_25_-P_75_:1.15 to 2.10), indicating no resistance development.

**FIG 3:**
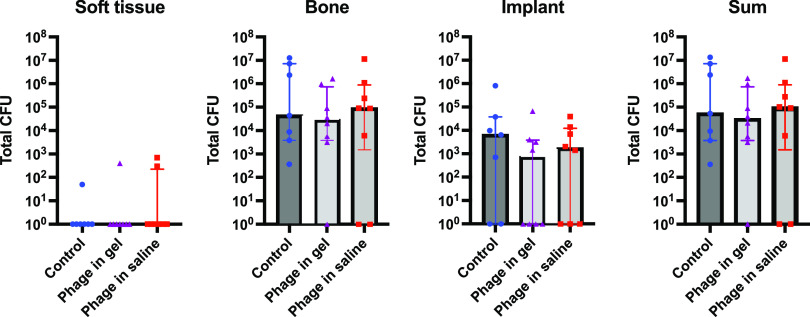
Quantitative bacteriological evaluation of soft tissue, implant and bone and the sum of all three is shown for the control animals receiving systemic antibiotics alone (*n* = 7), animals treated with antibiotics plus phage therapy through a subcutaneous access tube (*n* = 8) and animals treated with antibiotics plus phage-loaded hydrogel (*n* = 8). All animals received treatment 2 weeks after inoculation (i.e., established infection). The median and the interquartile range are shown for each group. No statistically significant (*P* < 0.05) results were found between groups. CFU, colony forming units.

**Treatment study.** The results from the treatment study with the rifampicin-resistant strain are displayed in Fig. 3. The bacterial load in the soft tissue was mostly eradicated in all groups, i.e., in 6/7 control animals, 7/8 animals that received the phage-loaded hydrogel and in 6/8 animals that received phage in saline. In the bone, no infection was found in 1/8 animals that received phage in gel and in 2/8 animals that received phage in saline. The bacterial load on the implant was eradicated in 2/7 control animals, in 4/8 animals that received the phage in gel and in 3/8 animals that received phage in saline. The infection was fully eradicated in one animal in the group that received the phage-loaded hydrogel (Group 4) and in two animals in the group that received phage in buffer (Group 3). In the control group receiving systemic antibiotic therapy (Group 5), all animals were infected at euthanasia. No significant differences were found in the rate of culture positive to negative or in bacterial load between groups.

The application of phage in saline via a subcutaneous access tube was complicated due to the superinfection of the site where the tube was inserted. All animals in the group receiving phage in saline showed pus surrounding the access tube and along the drain. However, cultures after sonication of the subcutaneous access tube and drain were negative in six of these animals. In two animals receiving phages through the tube, cultures were positive for pathogens other than S. aureus.

### Phage susceptibility and phage-neutralizing antibodies.

Aliquots of spleen, kidney, liver, bone, soft tissue, and implant were screened for the presence of ISP at euthanasia. However, ISP could not be recovered from any testing material after propagation with the inoculating S. aureus JAR060131 RifR strain.

**FIG 4:**
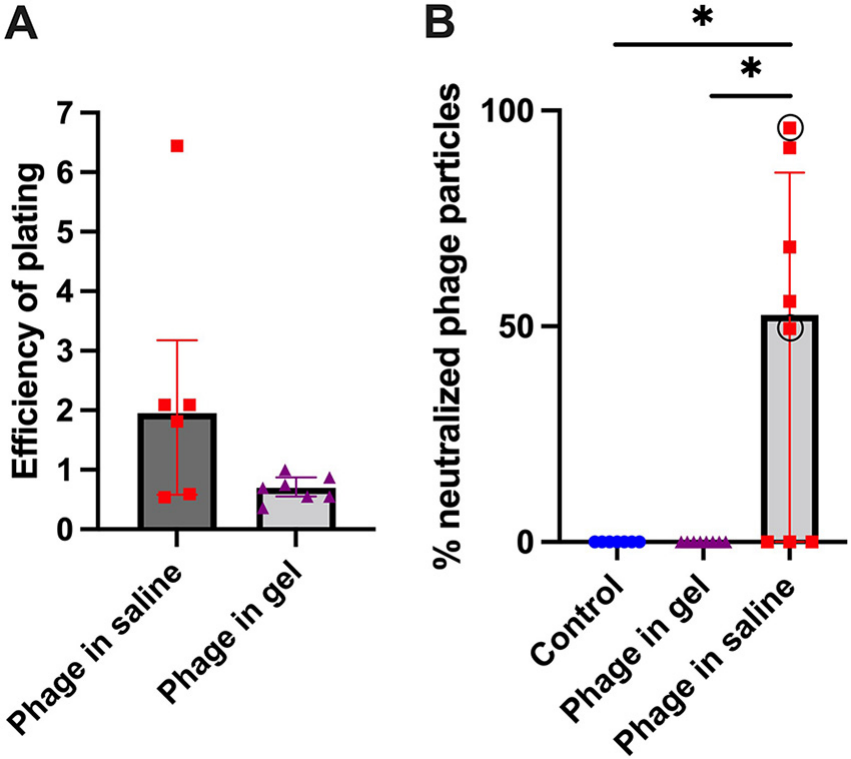
Phage susceptibility and neutralization tests. A. Efficiency of plating for the isolates retrieved after euthanasia of the animals in the treatment group. The median and interquartile range are plotted for each group. There was no statistically significant (*P* < 0.05) difference between the group receiving phage in saline or the group receiving phage in hydrogel. B. Percentage of neutralized phage particles. The differences between groups were statistically significant (*P* = 0.001), as indicated with an asterisk. For the group receiving phage in saline, a circle is drawn around the animals that were not infected at euthanasia.

Bacterial isolates retrieved after euthanasia from the groups that received phage therapy in the treatment study were tested for their susceptibility to the applied phage. The median EOP was 1.95 (P_25_-P_75_:0.58–3.18) for the group that received phage in saline and 0.7 (P_25_-P_75_:0.55–0.87) in the group that received the phage-loaded hydrogel, indicating that the retrieved isolates remained susceptible to ISP after treatment with phage therapy (Fig. 4A). There was no significant difference between groups.

The presence of phage neutralizing antibodies in plasma at euthanasia was tested in the treatment study (Fig. 4B). The mean percentage of neutralized phages in the group receiving phage in saline (Group 3) was 45% ± 40%. Five out of eight animals receiving phage in saline developed neutralizing antibodies. No phage neutralization could be found at euthanasia in the group that received the phage-loaded hydrogel (Group 4) or in the Control group receiving systemic antibiotics only (Group 5). This difference was statistically significant.

### Clinical observations.

**FIG 5:**
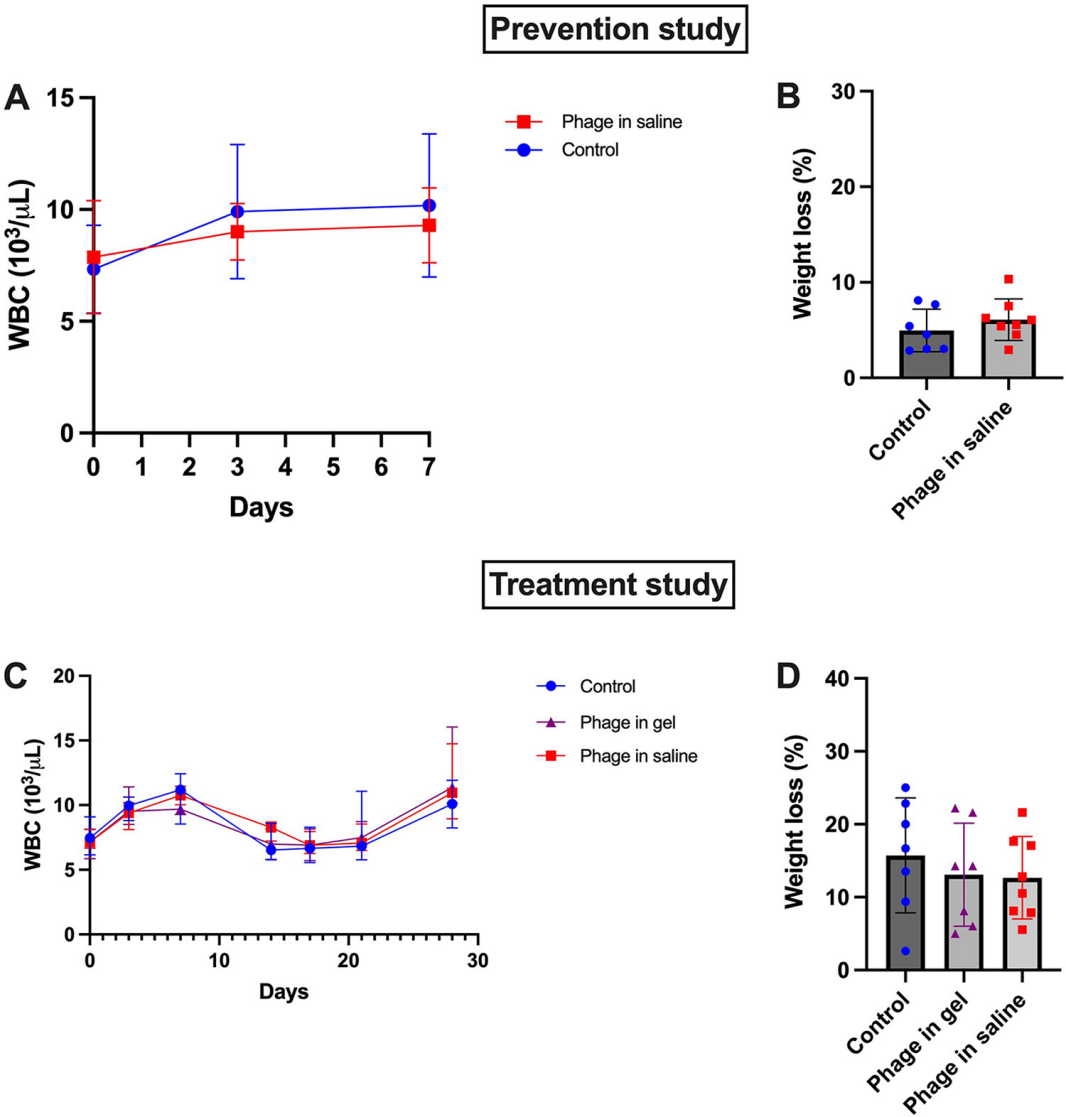
White blood cell counts and weight change in the prevention and treatment study. A. Mean white blood cell counts for the animals during the prevention study. The mean and standard deviation are plotted for each group. B. Percentage weight change between euthanasia and index surgery for the animals in the prevention study. The mean and standard deviation are plotted for each group. WBC: white blood cell count. C. Median white blood cell counts for the animals in the treatment study. The median and interquartile range are plotted for each group. D. Percentage weight change between euthanasia and index surgery for the animals in the treatment study. The mean and standard deviation are plotted for each group. WBC, white blood cell count.

In the prevention study, mean white blood cell (WBC) counts were within the normal range in both the phage treated and the control group throughout the study period (i.e., from index surgery until euthanasia) (Fig. 5A). In both groups, the mean WBC count at euthanasia did not significantly differ from the mean WBC count at the index surgery. The mean percentage weight change between euthanasia and index surgery is displayed for both groups in Fig. 5B. No significant differences were found comparing the WBC counts and weight changes between groups.

In the treatment study, the median WBC counts did not significantly differ between groups at index surgery and were within the normal range across all groups (Fig. 5C). In the control group (Group 5), there was no significant difference between the median WBC count at index surgery (day 0) and the median WBC count at euthanasia (day 28). In the group that received phage in saline (Group 3) and the group that received the phage-loaded hydrogel (Group 4), there was a significant difference (not shown) between the median WBC count at index surgery and the median WBC count at euthanasia. The median WBC counts at euthanasia did not differ significantly between groups. Considering the mean percentage weight change between the index surgery and euthanasia (Fig. 5D), no significant difference was observed between groups.

## DISCUSSION

From a therapeutic viewpoint, phage therapy can be considered a natural, specific and self-limiting strategy and is currently being revisited as a sustainable approach to target (multidrug) resistant bacteria and difficult-to-treat cases. Although several reports have shown promising clinical outcomes in patients with orthopedic device-related infections, (11, 12, 14, 16), questions remain regarding the optimal application protocol, systemic effects of phage therapy and the impact of the immune response, specifically phage-neutralizing antibodies (17, 23). This proof-of-concept study evaluated the application of phage therapy for the prevention and treatment of FRI in a clinically relevant rabbit model.

### Prevention study.

The intraoperative application of phage in saline was highly effective in preventing infection, as six out of eight animals (75%) were not infected at euthanasia. This compares favorably with the control group, in which only one out of seven animals (14%) was infection-free at euthanasia. To our knowledge, this is the only study that assessed the efficacy of phage therapy (in suspension [saline]) for the prevention of FRI caused by S. aureus in a clinically relevant fracture model. Two studies evaluated the use of phage therapy in a similar setting in murine models, but applied phages via local delivery systems, such as a polyethylene oxide hydrogel loaded with a phage cocktail against P. aeruginosa (24) and a hydroxypropyl methylcellulose coating containing a S. aureus phage and/or antibiotics (25). Both studies showed a statistically significant reduction in bacterial load compared to controls. Furthermore, in the study by Kaur et al., the use of a dual coated implant was more effective in reducing bacterial adherence compared to single coated or naked implants (25). The concomitant use of antibiotics with phages has been shown to work synergistically, resulting in better treatment outcomes (26). In fact, the local administration of antibiotics is gaining importance in the field of FRI for both prevention and treatment purposes. It may, therefore, be foreseen that the combination of phages and antibiotics should be investigated for direct application into surgical wounds. There may be synergistic benefits in the combination, as well as reduced risk of resistance developing. Bacterial resistance to phages generally involves cell surface receptor modifications, which hamper phage adsorption (27). Such modifications may thus lead to an increased susceptibility to antibiotics (28–30). On the other hand, some antibiotics that affect bacterial protein synthesis (such as aminoglycosides) have been shown to interfere with the phage life cycle, resulting in an attenuation of phage replication (31). Synergistic or antagonistic interactions between phages and antibiotics therefore depend on several factors, such as the class of antibiotic that is applied and its mechanism of bacterial inhibition, the receptor involved in phage adsorption and the order of treatment (i.e., simultaneous or sequential administration) (32–34). Further research is required to evaluate the extent and relevance of this phenomenon before phage therapy, alone or in combination with antibiotics, could become widely adopted in clinical practice (31).

The efficacy of phage in saline, as shown in our study, supports our initial hypothesis that prevention would require immediate release of phage and rapid distribution throughout the wound, whereas encapsulation of phage into a carrier – such as a hydrogel – functions to delay and control release. The phage-loaded hydrogel was therefore not tested in a prophylaxis setting. Indeed, in a prophylaxis setting, the bacterial inoculum is in planktonic mode, and rapid killing of bacteria before a biofilm may form, could be a relatively easier target. In the treatment setting, with biofilm, potentially fibrous tissue formation and less easy access to the surgical site, eradication of infection is clearly more challenging. Of course, in clinical reality, the target bacteria are not known in a prophylaxis setting. The clinical implementation of phage irrigation for prophylaxis would require phage cocktails to cover a broad range of potential contaminating microorganisms (based on local epidemiology). That is also the case, to a certain degree, for the local administration of antibiotics, for instance for the prevention of FRI after an open fracture where broad spectrum antibiotics may be used but may not cover all potential pathogens adequately. Nevertheless, our study supports the concept that phages could offer good protection in a prophylactic setting.

### Treatment study.

Infection was eradicated in one animal and in two animals of the groups that received the phage-loaded gel or phage in saline, respectively, while all animals in the control group were infected. However, these results were not statistically significant. No phages could be recovered from the dissected tissues in both treatment groups. This may potentially be attributed to the wash-out period of 1 week after cessation of treatment prior to euthanasia, which may have also allowed animals that were less infected at the end of treatment to relapse and regrow high numbers of bacteria.

### Phage in saline.

As stated above, no significant bacterial load reduction was found for animals that received phage in saline through a subcutaneous access tube. Furthermore, the application of phages was complicated due to the superinfection of the site where the access tube was inserted. That is, abscesses were found surrounding the tube, which were culture negative in six out of eight animals, while in two animals other bacterial species were isolated (other than S. aureus). This was not found in the group receiving the phage-loaded hydrogel, which again highlights the fact that the presence of an access tube or draining system is at risk of superinfection. This risk may be higher in a preclinical setting compared to a clinical setting where the conditions are more suitable to maintain sterility of the wound during patient care. To our knowledge, this is the first preclinical study that used a subcutaneous access tube to apply phage therapy. Previously published preclinical studies generally use repeated local injections of phage in suspension, either directly into the infected site (35) or intraperitoneally (36). Although these studies seem to show greater efficacy of phage therapy in treating implant-related bone infection, their route of administration (through repeated local administrations) does not seem clinically realistic. That is especially the case in patients with FRI or osteomyelitis, where the infection may be extensive, including an entire bone. A local delivery system (such as a hydrogel) may be more useful in such cases as it can be applied directly to the infected site and allows a sustained release. Furthermore, because such a delivery system only needs to be applied once intraoperatively, the risk of superinfection decreases.

### Neutralizing antibodies.

A major concern for the application of phage therapy is the induction of phage neutralizing antibodies (21). In our study, phage neutralizing antibodies were detected only in the group receiving phage in saline. In this group, a mean percentage of 45% ± 40% of phages were neutralized at plasma dilution 1:100. The intensity of anti-phage responses depends on several factors, such as the phage strain that is applied, with possible cross-reactivity between similar phages, the administration route and the overall immune status of the patient. Furthermore, higher phage titers and repeated administrations induce faster, stronger and more efficient anti-phage responses compared to a single administration (19).

The induction of phage neutralizing antibodies during initial phage therapy does not necessarily imply worse treatment outcomes (19, 20, 37). In fact, in our study, the animals that did not develop phage neutralizing antibodies were still infected. Plasma of two animals showed phage neutralization of more than 90%. One of these animals was not infected at euthanasia. We therefore cannot conclude that there is a correlation between our results and the development of phage neutralizing antibodies. An efficient and specific immune response also requires sufficient time (19). Several *in vivo* studies have shown that the neutralizing antibodies that were produced during the first phage administrations may cause a decrease in local phage titer during a second round of phage therapy and can thus potentially contribute to treatment failure (38). However, it is not known how this extrapolates to phage therapy in human subjects (19, 39). In a report of four patients with a musculoskeletal infection who were treated locally with phage therapy (including ISP) for seven to 10 days through an external draining system, no neutralizing phage antibodies were found during and up to 3 months after treatment (12), which may indicate a discrepancy between the human and rabbit immune response to phage treatment. Another study evaluated the antiphage activity in sera from 122 patients who were treated at the phage therapy unit in Wroclaw, Poland (20). These patients received phage therapy for a variety of infectious diseases, including bone infections, and causative pathogens. In this study, higher phage inactivation rates did not correlate with a worse clinical outcome, although since the antiphage activity of sera after phage therapy varied from patient to patient, it is difficult to generalize these results (20). Furthermore, the phages that were used were not purified (phage lysates were used), and phage therapy was continued for several weeks, which may have contributed to the results (20). This is supported by a recent case study by Dedrick et al. (40). They report on the intravenous administration of a phage cocktail in an 81-year-old patient with bronchiectasis and refractory Mycobacterium abscessus lung disease. After 2 months, the phage neutralizing immune response increased, resulting in limited therapeutic efficacy as M. abscessus counts increased (40). Interestingly, the same administration protocol was used in a 15-year-old immunocompromised posttransplant patient with cystic fibrosis (41). In this patient no phage neutralizing antibodies were found, and the infection was eradicated after phage treatment (41). While there are other differences between both case reports such as age and cystic fibrosis status, the most striking difference was the immune status (40).

These studies underline the fact that knowledge on phage neutralizing antibodies is still very limited and the extreme diversity of phages and administration protocols does not allow us to draw general conclusions (19, 20, 37, 39). To compensate for the effects of neutralizing antibodies, it may be useful to adjust dosing to avoid unnecessary exposure of the immune system to phages and to monitor the immune reaction during therapy (19). Furthermore, phage encapsulation in for instance a phage-loaded hydrogel may protect phages from (early) immune neutralization (10, 19, 21, 39, 42). Indeed, in our study, no phage neutralization was detected in the group that received the phage-loaded hydrogel. Further investigation is therefore warranted to fully understand the mechanisms underlying this as it may prove to be a key benefit of biomaterials-based encapsulation strategies to extend the window for phage efficacy in therapeutic applications.

### Phage-loaded hydrogel.

A possible trend of minor bacterial load reduction (1 log reduction in median CFU) was observed with the phage-loaded hydrogel on the implant (where the infection was eradicated in 4/8 animals), which was achieved in only 2/8 rabbits receiving antibiotics alone, although this was not statistically significant. Similar results were found by Cobb et al. who applied an alginate hydrogel loaded with a CRISPR-Cas9-modified S. aureus bacteriophage (10^7^ PFU/ml) in a rat model of implant-associated osteomyelitis (43). The bacterial load in the soft tissue was significantly reduced in the groups receiving phage therapy or antibiotic therapy, but the effect of phage treatment was comparable to that of antibiotics. However, this was not observed in the bone, as only a high dose of phosphomycin (3g) could significantly reduce the bacterial load (43). The authors attributed this to the relatively low phage titer in the hydrogel (10^7^ PFU/ml) (43). In general, phage loading inside biomaterials consists of higher phage titers compared to the phage titers used in recent phage treatment protocols applied in saline suspensions (12, 21). It is hypothesized that with higher titers within the biomaterials, a sustained release of adequate phage titers may be maintained resulting in prolonged therapeutic efficacy. However, evidence regarding the optimal or minimal effective phage titer is scarce and is subject to phage-specific as well as patient-specific variability (21). It may also be that in our study, the dual application of antibiotic and phage masked the contribution of each. We did not include a phage treatment only group, as clinical treatment, even with phage therapy, still includes standard of care antibiotic therapy. It could be that the antibiotic therapy reduced the bacterial load rendering fewer bacterial hosts to support propagation of phage within the wound. *In vitro* data suggest that when combining antibiotic and phage, it may be beneficial to apply the phage initially, and follow that with antibiotic therapy to ensure maximal effect (26, 44). Further development of the phage-loaded hydrogel may benefit from dual application, with controlled and staggered release of antibiotic and phage.

A recent case report describes the use of a phage-loaded hydrogel as a salvage procedure to treat a human patient with an infected knee megaprosthesis (15). During a DAIR (debridement antibiotics and implant retention) procedure, a Defensive Antibacterial Coating (DAC) hydrogel loaded with phages against S. aureus (10^9^ PFU/ml) was applied (15). This study suggests the practical feasibility of the application of a phage-loaded hydrogel carrier, especially in cases where a DAIR procedure is the only option and the prosthetic surface is large. Further research is required to assess the compatibility of different phages with a hydrogel carrier and its efficacy to eradicate the infection (15). Although the same phage titer of 10^9^ PFU/ml (as in the aforementioned case study) was applied in our study and the ISP was stable within the hydrogel (data not shown), we did not observe a statistically significant reduction of the bacterial load *in vivo*, compared to the control group. Furthermore, no phages could be recovered from the tissues (bone, soft tissue, implant or organs) suggesting that phage was not present in the tissue for an extended period of time. Embedding, encapsulating, or adsorbing phages into carriers offering controlled release over a longer period of time may therefore be beneficial in treating established infection. Further research, involving different phage types, concentrations, or delivery vehicles, is therefore advisable.

## CONCLUSIONS

This study provides a proof-of-concept regarding the application of phage therapy in a clinically relevant model for FRI. The intraoperative application of phage in saline prevented the development of FRI compared to control animals that only received antibiotic prophylaxis. However, further research is required to evaluate whether phage therapy can be applied in clinical prevention settings. In a treatment setting, phage therapy via a subcutaneous access tube approximates the clinical situation, but this approach was complicated by superinfection in our model and the development of phage neutralizing antibodies. Phage neutralization was not found in the plasma of the animals that received the phage-loaded hydrogel, which may indicate that encapsulation of phages into a carrier such as a hydrogel limits their exposure to the adaptive immune system. Further research and improvements of these application methods are required.

## MATERIALS AND METHODS

### Animal care and use, surgical procedure, and study design.

This study was approved by the Ethical Committee of the canton of Grisons in Switzerland (approval numbers 07_2019, 03E_2020, 14_2020). All procedures were performed in an AAALAC (Association for Assessment and Accreditation of Laboratory Animal Care International)-approved facility and according to Swiss animal protection laws and regulations. Skeletally mature specific pathogen-free (SPF) female New Zealand White rabbits (Charles River, Sulzfeld, Germany) between 20 and 32 weeks of age and with a mean body weight of 3.3 ± 0.8 kg (range 3.0 kg to 4.1 kg) were used in this study. All animals were screened prior to entry into the study and found to be healthy after a clinical examination and evaluation of the hematocrit and WBC count (Vet ABC, Scil animal care, Viernheim, Germany). All animals were allowed to acclimatize to their surroundings for 2 weeks prior to the start of the study. During this time, they were group housed with a 12h dark – 12h light cycle, fed with hay, lettuce and supplemental feed for rabbits (Granovit AG, Lucens, Switzerland).

**FIG 6:**
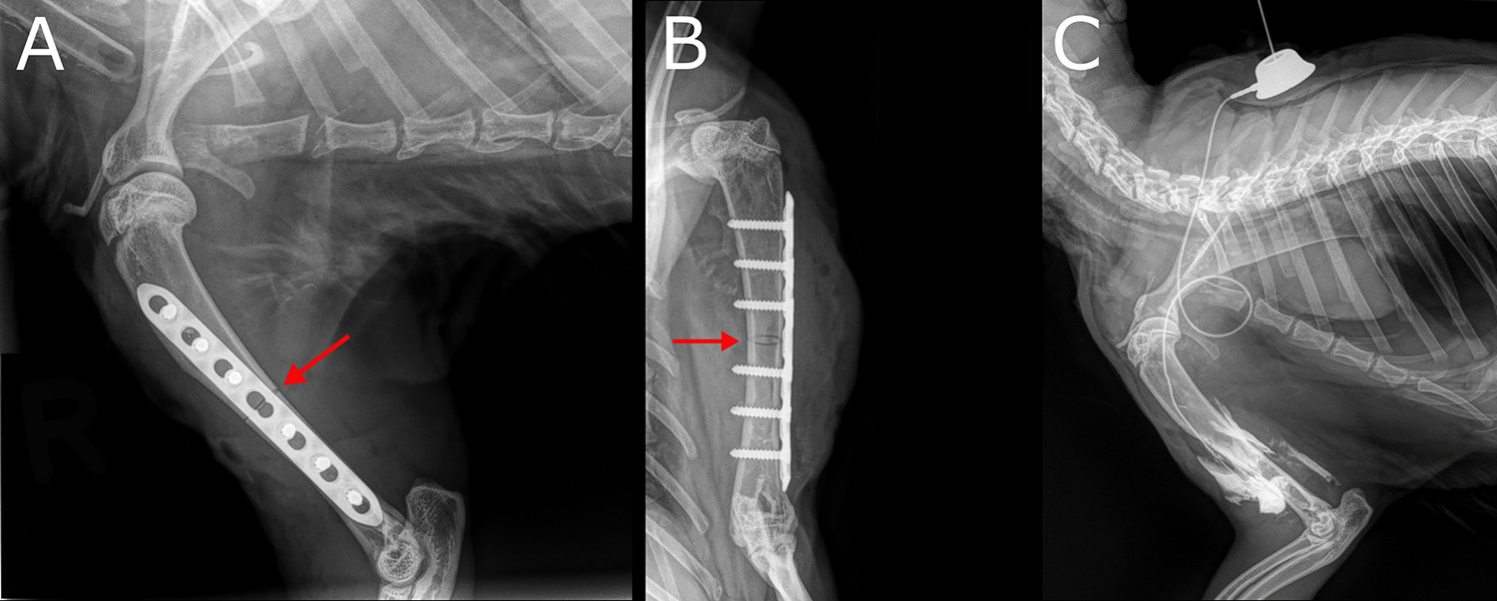
Radiological images of the rabbit humerus fixation, osteotomy and subcutaneous access tube. A. Postoperative, lateral radiograph of the right humerus with the plate and osteotomy (red arrow) *in situ*. B. Anteroposterior radiograph of the right humerus with the plate and the osteotomy (red arrow) *in situ*. C. Latero-medial radiograph of the right humerus of a rabbit during a cadaver trial with the subcutaneous access tube *in situ* which was later used for phage delivery (see main text). In this cadaver trial, 1 ml of contrast medium was directly injected into the peri-implant space via the subcutaneous access tube to evaluate the distribution of the injected fluid. The injection port with the inserted needle is placed between the shoulder blades. The tube is in contact with the osteotomy area. The more opaque zones surrounding the humerus can be attributed to the injected contrast medium.

The study design is displayed in Fig. 1. All animals underwent index surgery, including a humeral osteotomy and plate osteosynthesis (Fig. 6A and B). The animal experiments were performed in two phases. First, we tested phage therapy in a prevention setting (Fig. 1A), whereby bacterial inoculation and phage delivery were applied during the same procedure. All animals received antibiotic prophylaxis, to simulate current clinical practice. Test groups received either phage in saline (Group 1) or no treatment (Group 2), with a target of eight rabbits per group. In the second phase of this study, we assessed the ability of phage therapy to support conventional antibiotic therapy of an established infection (Fig. 1B). The same animal model was used as in the prevention study, but prophylactic antibiotics were omitted and the bacterial infection was allowed to develop for 2 weeks before treatment commenced. Treatment groups included phage in saline with concomitant antibiotic therapy (Group 3), a phage-loaded hydrogel with concomitant antibiotic therapy (Group 4), and antibiotics only (Group 5), with a target of eight rabbits per group. Group sizes were calculated in both studies based on an effective difference of 80% (i.e., an infection rate reduction of 80%). For all groups, outcome was measured by quantitative bacteriology, radiographic evaluation and blood work, including WBC count and the detection of phage neutralization caused by phage-neutralizing antibodies in blood plasma.

Surgery was performed as described previously by Arens et al. (45). In summary, a middiaphyseal osteotomy was created with a 0.44 mm Gigly saw (RISystem AG, Landquart, Switzerland) and fixed with a seven-hole locking plate and six 2 mm locking screws. Inoculation was performed by pipetting 34 µl of bacterial suspension of S. aureus JAR060131 onto the central screw hole overlying the osteotomy and to the adjacent proximal and distal screw holes.

In the prevention study (Fig. 1A), all animals received a single shot of cefuroxime (Sandoz AG, Basel, Switzerland) intravenously, at a dosage of 18.75 mg/kg (weight-adjusted dosage based on human medicine for a nominal human with a bodyweight of 80 kg receiving a standard dosage of 1500 mg) (46). In the animals that received phage therapy (Group 1), the first layer was temporarily closed after inoculation with a 4.0 Monocryl suture (Ethicon Inc., NJ, USA). After 5 min, the sutures were reopened to apply 6 ml of phage ISP in normal saline at a phage titer of 10^8^ PFU/ml. A contact time of 15 min was allowed, after which the remaining volume was removed with a syringe and the wound was closed. In the control animals (Group 2), the incisions were closed after inoculation. All animals in the prevention groups were euthanized after 1 week.

In the treatment study (Fig. 1B), the infection was allowed to establish for 2 weeks, after which all rabbits underwent revision surgery to excise visibly necrotic tissue and flush out the wound with saline. In the animals in Group 3, a subcutaneous access tube (CP-100K – Le Petite kit, Norfolk Vet Products Inc.) was placed intraoperatively in the subcutaneous layer between the shoulder blades. The tube end was placed in contact with the osteotomy and anchored with a Chinese finger trap technique to the fascia (Fig. 6C). After the placement of the subcutaneous access tube, 6 ml of phage in normal saline (ISP titer of 10^8^ PFU/ml) was applied to the surgical site. A contact time of 15 min was allowed, after which the remaining fluid was collected with a syringe. In Group 4, 800 µl of phage-loaded hydrogel (ISP titer of 10^9^ PFU/ml) was applied on the surface of the plate, after which the wounds were closed. The control animals (Group 5) only underwent debridement and irrigation of the wound, after which the incisions were closed. All animals in the treatment groups (Groups 3 – 5) received nafcillin subcutaneously (40 mg/kg four times per day) and rifampicin orally (40 mg/kg two times per day) for 7 days after the revision surgery. During this week, the animals of Group 3 received phage suspended in normal saline as 1 ml injections (10^8^ PFU/ml) directly into the infected site via the subcutaneous access tube, two times per day. After a subsequent 1-week wash-out period with no antibiotic or phage treatment, all animals were euthanized and bacterial analysis was performed.

### Animal welfare.

After the initial surgery, the animals were single-housed for the entire duration of the study. All animals were checked daily by a veterinarian or experienced animal caretaker. Blood samples were taken preoperatively, 3 days postoperatively and weekly thereafter until the end of the study period. The WBC count was determined (Vet ABC, Scil animal care, Viernheim). Blood plasma (micro tube 1.3 ml K3E 1.6 mg EDTA/ml, Sarstedt AG, Nümbrecht, Germany) was collected through the ear vein at the same time points and stored at −20°C for the phage neutralization assay. Weight was measured at surgery, after 3 days and weekly thereafter. Radiographs of the operated limb were taken in two planes after each surgery to check alignment and fixation, and at the end of the study to check for radiographic signs of infection. After the study period, all animals were humanely euthanized using intravenously administered pentobarbital (Esconarkon, Streuli pharma AG, Uznach, Switzerland).

### Bacteria and inoculum preparation.

The clinical S. aureus strain JAR 060131 (CCOS number 890, Culture collection of Switzerland, Wädenswil, Switzerland), isolated from a patient with an orthopedic device-related infection, was used for preparation of the inoculum. This strain was used for the prevention study and was susceptible to cefuroxime, which was used as antibiotic prophylaxis. The treatment study was initially set up using the same bacterial strain. However, antibiotic treatment was effective in seven out of eight control animals (88%, results not shown). To have a better model to evaluate the effects of phage therapy in a treatment setting, this experiment was repeated with a rifampicin-resistant isolate of the initial strain. To generate the rifampicin-resistant mutant, JAR060131 strain was cultured in Müller-Hinton broth (MHB, Oxoid Ltd., Hampshire, United Kingdom) containing rifampicin (Sigma-Aldrich, St. Louis, MO, USA) at concentrations ranging from 0.25 to 256 mg/liter. Bacterial suspensions were incubated statically at 37°C for 24 h. Next, the highest concentration with visible bacterial growth was recultured (strain here named JAR060131RifR) in fresh MHB without antibiotics. Frozen glycerol stocks (MHB containing 20% glycerol) were prepared and stored at −20°C for further examinations. Resistance to rifampicin was confirmed by the disk diffusion zone of inhibition (ZOI) method following the EUCAST instruction, using 5 µg rifampicin disks (Oxoid Ltd., Hampshire, United Kingdom). The ZOI was measured directly after generating rifampicin resistance, and after 1, 4 and 24 weeks recovery from frozen storage. Stability of rifampicin resistance in JAR-060131RifR was further checked after serial passage (10 times) from one antibiotic-free MHA plate to a fresh plate. Each time, the zone of inhibition from a single colony was measured and was 0 mm compared to the parent control strain JAR060131, which had a 34/35 mm ZOI indicative for a stable rifampicin resistance in JAR060131RifR. Furthermore, this strain was susceptible to nafcillin, which was used in combination with rifampicin in the treatment study.

One day before surgery, an overnight culture (rifampicin sensitive or resistant, as appropriate) was prepared by suspending a single bacterial colony in 50 ml Tryptic Soy Broth (TSB; Oxoid Ltd., Hampshire, United Kingdom) at 37°C while shaking. From the overnight culture, a fresh logarithmic phase culture was prepared in TSB. Bacteria were washed twice in Phosphate buffered saline (PBS, Sigma-Aldrich, St. Louis, MO, USA) 1.5h prior to surgery and diluted in PBS to obtain a target inoculum of 2.0 × 10^6^ CFU (CFU) of S. aureus. Immediately after inoculum preparation, quantitative cultures were performed by plating a dilution series to ensure the accuracy of the used inocula.

### Bacteriophage therapy.

ISP, a monophage targeting S. aureus was used with permission from the Eliava Institute, Georgia. This phage has been characterized in detail: its genetic sequence and therefore the confirmation of its strictly lytic profile and absence of toxins or antibiotic resistance genes is available (47, 48). Phage ISP has a contractile tail and belongs to the *Kayvirus* genus (*Herelleviridae*, *Twortvirinae*). ISP is commonly applied therapeutically in patients and has also been used in preclinical research (12, 49). The ability of ISP to infect S. aureus JAR060131 was tested using the spot test and double agar overlay method as previously described (50, 51).

### Preparation of phage-loaded hydrogel.

All reagents for the preparation of the emulsion-based hydrogel (EBH) were purchased from Sichma Aldrich, St. Louis, MO, USA. EBH was prepared 7 days prior to application by mixing homogeneity soy phospholipids (1.2%) with soybean oil (10%), glycerin (2.3%) and water for injection (64%) with a turbomixer. The homogeneous mixture was added to carboxymethyl cellulose (3%) followed by passive hydration overnight and sterilization with steam for 20 min. On the day of revision surgery, 100 µl of ISP phage (stock titer 10^10^ PFU/ml) was mixed with 100 µl SM phage buffer (10 mM Tris, 10 mM MgSO_4_, 150 mM NaCl, adjusted to pH 7.5) and 800 µl of EBH gel. Of this suspension, 800 µl was pipetted onto the plate intraoperatively.

### Bacterial quantification.

After euthanasia, the humerus was harvested. Soft tissue, abscesses and implants were collected and cultured. Soft tissue overlying the plate was homogenized (Omni TH, tissue homogenizer TH-02/TH21649) in 5–20 ml PBS (Sigma-Aldrich, St. Louis, MO, USA). The plate and screws were removed from the humerus, transferred in a glass test tube containing 10 ml PBS and sonicated in an ultrasonic water bath (Model RK 510 H, Bandelin electronic GmbH & Co. KG, Berlin, Germany) for 3 min. The humerus itself was dissected as a whole and homogenized (Polytron System PT 3100, Kinematica Ag, Switzerland) in 20–40 ml PBS. Serial dilutions were made for each sample, which were cultured on 5% horse blood agar plates (BA, Oxoid Ltd., Hampshire, United Kingdom). Plates were incubated at 37°C and CFU were quantified after 24 and 48 h. If no colonies grew on BA plates after 24 h of incubation, the entire sample (stored overnight at 4°C) was passed through a membrane filtration system (EZ-Fit^TM^ Merk KGaA, Darmstadt, Germany) and the filter membrane was incubated on BA plates for an additional 24 h at 37°C. All agar plates were kept an extra 24 h at room temperature and were checked for any signs of slow-growing colonies or contaminants. Irrigation fluids were therefore considered to have a lower limit of detection of one CFU per total collected volume.

Bacterial growth was confirmed as S. aureus using a latex agglutination test (Staphaurex, Remel Europe Ltd., United Kingdom). Resistance to rifampicin was confirmed by the disk diffusion ZOI method following the EUCAST instructions using 5 µg rifampicin disks (Oxoid Ltd., Hampshire, United Kingdom).

### Phage susceptibility tests.

In the treatment group, each isolated pathogen at euthanasia was tested for susceptibility against ISP. The EOP was determined, which is defined as the number of PFU a phage is able to produce when incubated with the bacterial strain isolated at euthanasia relative to the number of PFU the phage is able to produce when incubated with the initial (reference) bacterial strain.

### Phage titration and phage neutralization assay.

In all animals that were treated with phage therapy, the presence of phages in the bone, soft tissue, surrounding the implant and in the organs (i.e., spleen, liver, kidneys) was measured at euthanasia according to the protocol described by Dufour et al. (52). The spleen, liver and kidneys were dissected as these are considered the major organs responsible for phage clearance, and homogenized (Omni TH, tissue homogenizer TH-02/TH21649) in 5–20 ml PBS (Sigma-Aldrich, St. Louis, MO, USA). An aliquot of each tissue sample was obtained and centrifuged at maximum speed for 5 min at 4°C to pellet cells and debris. The supernatant was then filtered (0.45 µm, Millex, Merck Millipore, Ireland). Serial dilutions were made in SM phage buffer followed by phage titrations using the double agar overlay method, as previously described (12). The JAR 060131 strain was used to perform titrations.

To determine if phage neutralization occurred by the development of neutralizing antibodies in the rabbits during treatment, phage neutralization by plasma was performed using the modified Adams protocol, as previously described (12). Samples were diluted 1:100 in normal saline at 37°C. 900 µl of diluted serum was incubated with 100 µl of phage (titer 10^7^ PFU/ml) for 30 min at 37°C. After this time interval this mixture was diluted 100 times in cold normal saline and phage titration was performed using the double-agar overlay method.

### Data analysis.

Data were analyzed using SPSS statistics Version 27 (IBM, Chicago, IL, USA). Normality of continuous data were tested with the Shapiro-Wilk test and homogeneity of variances was tested using the Levene’s test. In case of parametric data, a one-way ANOVA or Student's *t* test (with either equal variance assumed or not) was used to compare differences between groups. In case of nonparametric data, the Kruskal-Wallis or Mann-Whitney U test was used, as appropriate. Paired data were evaluated using the paired samples *t* test or the Wilcoxon rank sum test, as appropriate. *P values* below 0.05 were considered significant. Data were visualized using GraphPad Prism 8 (GraphPad Software, San Diego, CA, USA).
